# Efficacy of Modafinil on Fatigue and Excessive Daytime Sleepiness Associated with Neurological Disorders: A Systematic Review and Meta-Analysis

**DOI:** 10.1371/journal.pone.0081802

**Published:** 2013-12-03

**Authors:** Ping Sheng, Lijun Hou, Xiang Wang, Xiaowen Wang, Chengguang Huang, Mingkun Yu, Xi Han, Yan Dong

**Affiliations:** 1 Department of Neurosurgery, Shanghai Institute of Neurosurgery, Military Institute of Neurosurgery, Changzheng Hospital, Second Military Medical University, Shanghai, China; 2 Department of Neurosurgery, Huashan Hospital, Fudan University, Shanghai, China; 3 Neuroscience Center, Changzheng Hospital, Second Military Medical University, Shanghai, China,; Charité University Medicine Berlin, Germany

## Abstract

**Background:**

Modafinil is a novel wake-promoting agent approved by the FDA ameliorating excessive daytime sleepiness (EDS) in three disorders: narcolepsy, shift work sleep disorder and obstructive sleep apnea. Existing trials of modafinil for fatigue and EDS associated with neurological disorders provided inconsistent results. This meta-analysis was aimed to assess drug safety and effects of modafinil on fatigue and EDS associated with neurological disorders.

**Methods:**

A comprehensive literature review was conducted in order to identify published studies assessing the effects of modafinil on fatigue and EDS associated with neurological disorders. Primary outcomes included fatigue and EDS. Secondary outcomes included depression and adverse effects.

**Findings:**

Ten randomized controlled trials were identified including 4 studies of Parkinson’s disease (PD), 3 of multiple sclerosis (MS), 2 of traumatic brain injury (TBI) and 1 of post-polio syndrome (PPS). A total of 535 patients were enrolled. Our results suggested a therapeutic effect of modafinil on fatigue in TBI (MD -0.82 95% CI -1.54 - -0.11 *p*=0.02, I^2^=0%), while a beneficial effect of modafinil on fatigue was not confirmed in the pooled studies of PD or MS. Treatment results demonstrated a clear beneficial effect of modafinil on EDS in patients with PD (MD -2.45 95% CI -4.00 - -0.91 *p*=0.002 I^2^=14%), but not with MS and TBI. No difference was seen between modafinil and placebo treatments in patients with PPS. Modafinil seemed to have no therapeutic effect on depression. Adverse events were similar between modafinil and placebo groups except that more patients were found with insomnia and nausea in modafinil group.

**Conclusions:**

Existing trials of modafinil for fatigue and EDS associated with PD, MS, TBI and PPS provided inconsistent results. The majority of the studies had small sample sizes. Modafinil is not yet sufficient to be recommended for these medical conditions until solid data are available.

## Introduction

Excessive daytime sleepiness (EDS) and fatigue are frequently encountered symptoms in neurological practice, which may arise from a variety of disorders such as Parkinson’s disease (PD), multiple sclerosis (MS), Alzheimer’s disease (AD), depression, stroke and traumatic brain injury (TBI) [1-13]. EDS is defined as not being able to keep alert or awake in daytime hours and might fall asleep in inappropriate situations. Different definitions have been proposed for fatigue. For example, in MS, fatigue has been defined as “A subjective lack of physical and/or mental energy that is perceived by the individual or caregiver to interfere with usual and desired activities” [14]. In general, fatigue is considered as a subjective feeling of tiredness, weakness, or lack of energy [15]. Fatigue and EDS not only severely impair productivity and performance, but may also have detrimental effects on social functioning and overall quality of life. The mechanisms of fatigue and EDS remain poorly defined, which seem to be multifactorial, arising from primary diseases related factors and other secondary ones. Recently growing evidence indicates that sleep disturbances, which are common in MS patients, may be an important contributing factor and treatment of sleep disturbances can improve fatigue and EDS in patients with MS [16-20]. Therapies for fatigue and EDS should address causal mechanisms if possible. Unfortunately, the potential mechanisms of fatigue and EDS in clinical practice are often hard to be understood and many factors may be involved. Hence, both pharmacological and non-pharmacological therapies have been applied in the management of fatigue and EDS [21-24]. 

Modafinil is a novel wake-promoting agent that is pharmacologically different from those of amphetamine and methylphenidate, the two classical psychostimulants. Its exact mode of action remains unclear. Modafinil may promote wakefulness through activation of noradrenergic and dopaminergic systems, probably through interaction with the hypocretin/orexin system [25,26]. Modafinil ameliorates EDS in all three disorders， i.e. narcolepsy, shift work sleep disorder (SWSD) and obstructive sleep apnea (OSA), and has been approved by the FDA [27,28]. Of note, the European Medicines Agency has recently recommended the use of Modafinil be restricted to the treatment of narcolepsy due to severe psychiatric side effects and skin reactions [29]. Furthermore, modafinil has been used in investigational treatment of EDS and fatigue associated with PD, MS, AD, stroke, TBI and post-polio syndrome (PPS) [30-46]. However, existing trials of modafinil for these neurological disorders provided inconsistent results. Although there have been some clinical trials on the effect of modafinil on fatigue and EDS associated with psychiatric disorders, such as attention deficit hyperactivity disorder, depression, schizophrenia and cocaine addiction, they are beyond the scope of the present study.

The current study employed meta-analysis to integrate the available literature on the treatment of modafinil on fatigue and EDS associated with neurological disorders and assessed the efficacy of modafinil on fatigue and EDS and its safety in patients with neurological diseases with a rigorous methodological quality assessment.

## Methods

### Selection of Studies

A comprehensive literature review based on Ovid Medline, EMBASE, the Cochrane and PSYCHInfo databases was conducted to identify published studies on the effect of modafinil on fatigue and EDS associated with neurological disorders. Search terms used were listed in supplement S1. The search was limited to articles written in English and published in peer-reviewed journals from January 1980 to December 2012. Studies must involve human subjects and primary data must be presented. Reference lists from the relevant studies were searched for additional literature.

### Inclusion criteria

Original studies were considered for inclusion in the meta-analysis if they met with the following criteria: (1) they were randomized controlled trials (RCT); (2) patients over 18 years old with neurological diseases such as PD, AD, MS, stroke, TBI, PPS and brain tumor were investigated; (3) the efficacy of modafinil on fatigue and EDS was examined; (4) results were sufficient to allow calculation of effect sizes.

### Data extraction and quality assessment

Two authors (PS and LJH) independently reviewed the full manuscripts of eligible studies. Data were extracted in standardized data-collection forms. Extracted data included first author’s name, year of publication, sample size, patients’ characteristics (mean age, gender), duration of treatment, dosage, type of disease, duration of disease, outcomes, baseline findings, country, study design and Jadad score. Any discrepancy was resolved by discussion with a third author (XH). Selected RCTs were critically appraised using the Jadad scale, which assesses the methodology of the study such as randomization (2 points), blinding (2 points) and attrition information (1 point) [47]. 

### Study outcomes

Primary outcomes included self-reported fatigue, which is then measured by single item scale and questionnaire instruments, as well as subjective EDS measured by Epworth Sleepiness Scale and objective EDS measured by Multiple Sleep Latency Test (MSLT) or Maintenance of Wakefulness Test (MWT). Secondary outcomes included depression and adverse effects.

### Statistical analysis

For dichotomous data, the impact of the intervention was expressed as relative risk (RR) with 95% confidence intervals (CI) using the Mantel-Haenszel method. For continuous data, the difference in change from baseline to follow-up between intervention and control groups was expressed as mean differences with 95% CI (if the same scale was used in all studies) or standardized mean differences with 95% CI (when different scales were used) using inverse variance method otherwise. Heterogeneity of treatment effects between studies was statistically explored by the I^2^ statistic, in which 0%–40% indicates unimportant heterogeneity, 30%–60% indicates moderate heterogeneity, 50%–90% indicates substantial heterogeneity, and 75%–100% indicates considerable heterogeneity [48]. The sensitivity analyses were carried out by excluding studies successively. All reported *P* values were two-sided, and *P* values less than 0.05 were deemed as statistically significant. The publication bias was statistically examined using the Egger’s regression model, calculated by Stata 12.0 (Stata Corporation, College Station, TX, USA).

## Results

### Study characteristics

**Figure 1:**
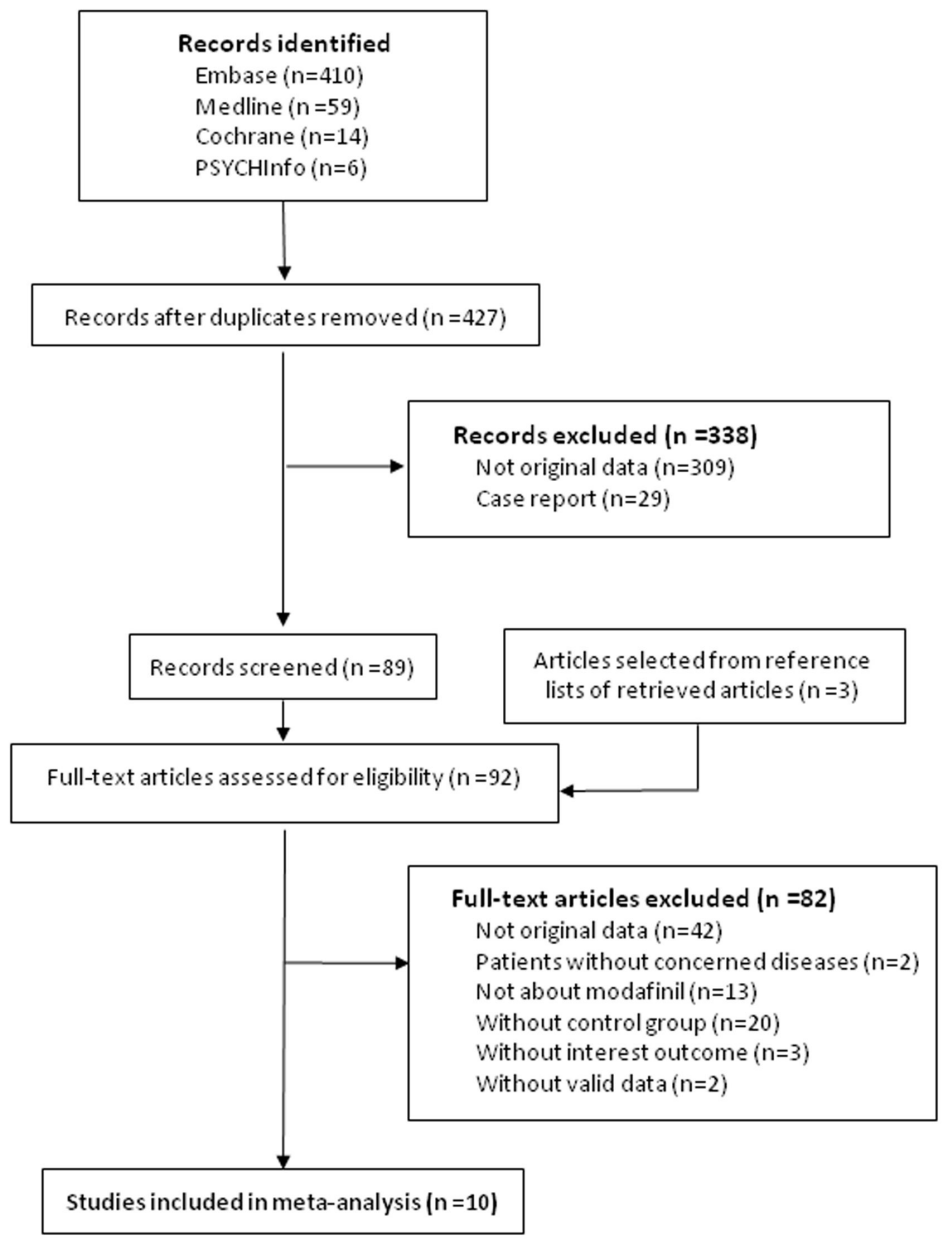
Flow diagram of the literature search and study selection processes.

A total of 427 citations were identified from the electronic searches and 3 through other sources, of which 338 were excluded after a preliminary review. The remaining 92 studies were retrieved for detailed assessment. Ultimately, 10 RCTs met the inclusion criteria (Figure 1). All studies were of good quality with a score of 3 or more assessed by the Jadad scale. Of 10 studies, 4 were identified in Parkinson’s disease, 3 in multiple sclerosis, 2 in traumatic brain injury and 1 in post-polio syndrome. Two studies were multi-centered in one country and the other 8 were at a single center. All studies were double-blinded and 4 were of crossover design. The included studies consisted of 535 patients with various sample sizes ranging from 19 to 110 (Table 1). 

**Table 1:** Design and patient characteristics for studies included in the meta analysis.

**Source**	**Samplesize** modafinil placebo		**Sex (male)**	**Mean age (year)**	**Duration of treatment (week)**	**Maximum dosage (mg/d)**	**Type of disease**	**Duration of disease (year)**	**Outcomes**	**Baseline findings (mean±SD, modafinil/placebo)**	**Country**	**Study design**	**Jadad score**
Lou JS (2009)	9	10	74%	67	8	200	PD	6.1	EDS	ESS: 8.3±4.8 / 9.8±4.7	USA	parallel	3
									Depression	CES-D: 17.1±10.5 / 23.6±10.4			
Ondo WG (2006)	19	18	73%	65	4	400	PD	6.8	Fatigue	FSS: 4.2±1.6 / 4.1±1.4	USA	parallel	5
									EDS	ESS: 15.7±3.1 / 16.0±4.8			
										MSLT: 6.4±5.1 / 4.5±3.9			
									Depression	HDS: 6.5±5.0 / 7.2±5.2			
Högl B (2002)	12	12	75%	65	2	200	PD	6.8	EDS	ESS: 13.2±2.2 / 11.8±3.8	Austria	crossover	4
									Depression	BDI: 9.6±6.9 / 10.1±7.3			
Adler CH (2002)	20	20	70%	65	3	200	PD	7.4	Fatigue	FSS: 4.4±1.5 *	USA	crossover	5
									EDS	ESS: 17.8±4.2 / 16.0±4.2			
Möller F (2011)	55	55	30%	41	8	200	MS	6.9	Fatigue	FSS: 6.01±0.75 / 5.8±0.75	Germany	parallel	4
										MFIS: 54.75±13.32 / 51.20±11.80			
									EDS	ESS: 11.80±4.89 / 11.78±4.96			
Lange R (2009)	9	10	12%	43	8	200	MS	8.1	Fatigue	FSS: 6.3±0.7 / 5.8±0.9	Germany	parallel	4
Stankoff B (2005)	48	57	32%	53	5	400	MS	>0.5	Fatigue	MFIS: 63.3±10 / 63.1±9.3	France	parallel	5
									EDS	ESS: 10.6±4.8 / 9.7±5.5			
Kaiser PR (2010)	10	10	85%	40	6	200	TBI	1.9	Fatigue	FSS: 4.6±0.8 / 5.0±1.4	Switzerland	parallel	5
									EDS	ESS: 10.0± 4.2 / 8.2±3.7			
										MWT: 15.6±11.4 / 18.7±9.3			
									Depression	BDI: 9±6 / 11±9			
Jha A (2008)	47	48	69%	38	10	400	TBI	5.8	Fatigue	FSS: 4.6±1.5 / 4.5±1.6	USA	crossover	5
										MFIS: 43.1±20.0 / 41.7±16.7			
									EDS	ESS:8.8±5.1 / 8.3±4.8			
									Depression	BDI: 18.5±7.9 / 18.5±7.9			
Vasconcelos OM	33	33	36%	61.05	6	400	PPS	15.8	Fatigue	FSS: 5.5±1.0 / 5.5±1.3	USA	crossover	5
(2007)									EDS	ESS:8.1±3.8 / 8.4±5.7			
									Depression	BDI: 10.5±8.0 / 8.8±6.7			

* For both modafinil and placebo groups.

Abbreviations: PD = Parkinson’s Disease; MS = Multiple Sclerosis; TBI = Traumatic Brain Injury; PPS = Post-polio syndrome; FSS = Fatigue Severity Scale; MFIS = Modified Fatigue Impact Scale; EDS = Excessive Daytime Sleepiness; ESS = Epworth Sleepiness Scale; MSLT = Multiple Sleep Latency Test; MWT = Maintenance of Wakefulness Test; HDS = Hamilton Depression Scale; BDI = Beck Depression Inventory; CES-D = Center of Epidemiological Study-Depression Scale

### Efficacy of modafinil on fatigue associated with neurological disorders

**Figure 2:**
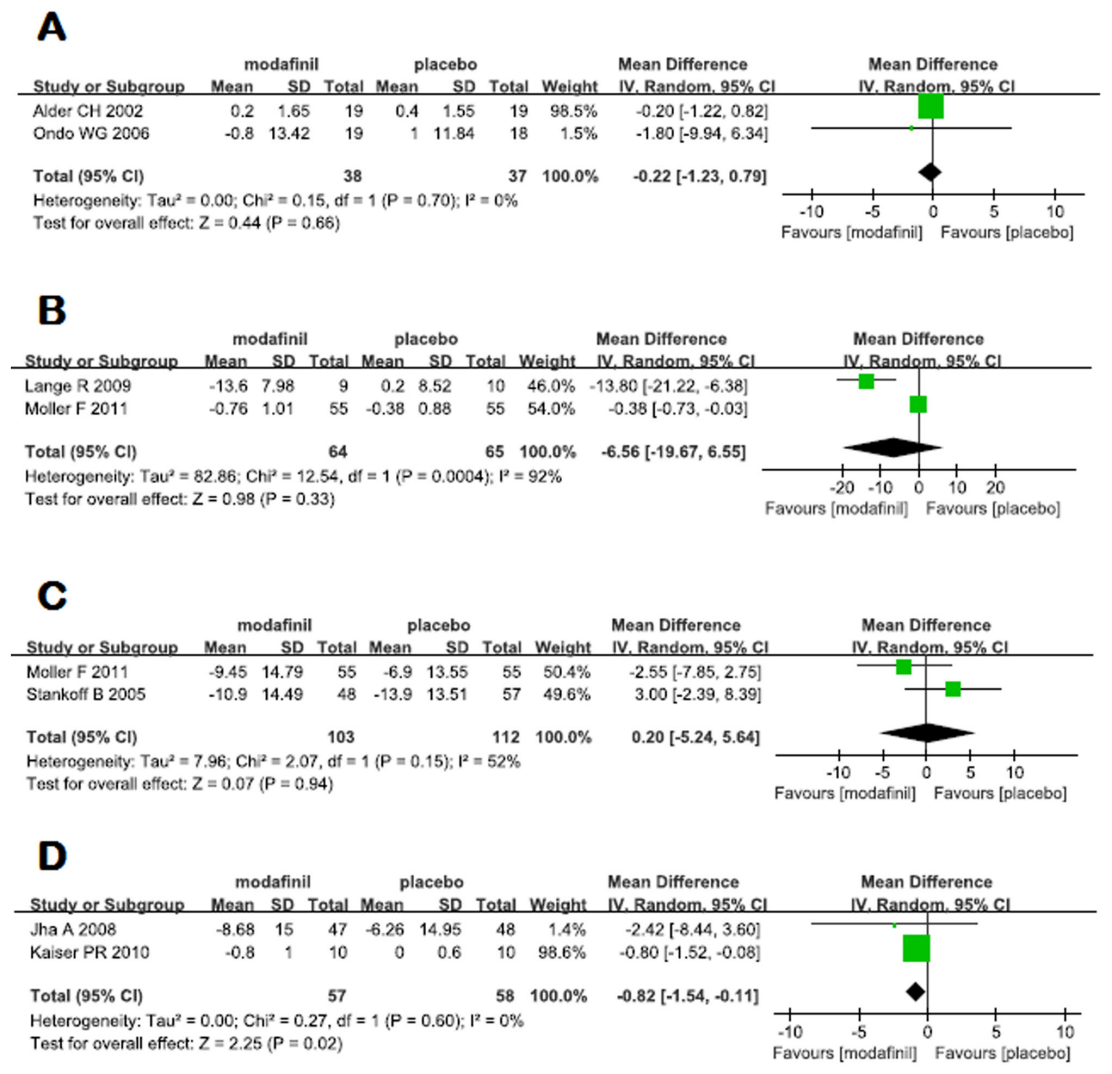
Effects of modafinil on fatigue in PD (A), MS (B) and TBI (D) measured by Fatigue Severity Scale and in MS (C) measured by Modified Fatigue Impact Scale.

Eight RCTs were included to investigate the effect of modafinil on fatigue associated with neurological disorders, with 2 studies on PD, 3 on MS, 2 on TBI and 1 on PPS, with heterogeneous outcomes among each other. Fatigue Severity Scale (FSS) was used in 2 studies of PD, with a pooled mean of -0.22 (95% CI -1.23 - 0.79), suggesting no significant effect of modafinil on fatigue associated with PD (*p*=0.66) (Figure 2A). Three studies were available for meta-analyses comparing modafinil in MS with placebo. FSS was employed in 2 studies and Modified Fatigue Impact Scale (MFIS) in 2 studies as well. Meta-analyses of fatigue measured by FSS and MFIS both failed to prove a beneficial effect of modafinil on fatigue associated with MS (-6.56, 95% CI -19.67 - 6.55, *p*=0.33, I^2^=92% for FSS; 0.20, 95% CI -5.24 - 5.64, *p*=0.94, I^2^=52% for MFIS) (Figure 2B and C). For TBI, 95 and 20 participants were included in the study of Jha et al and Kaiser et al, respectively. Meta-analysis of these two studies showed a therapeutic effect of modafinil on fatigue associated with TBI, with a mean difference of -0.82 (95% CI -1.54 - -0.11 *p*=0.02, I^2^=0%) (Figure 2D). Vasconcelos OM et al. conducted an RCT to investigate the effect of modafinil on fatigue associated with PPS, in which improvements were seen in FSS with both placebo and modafinil without significant differences between the two groups [46]. Owing to a limited number of trials, it was not possible to assess the presence of publication bias for each type of neurological disorders.

### Efficacy of modafinil on EDS associated with neurological disorders

**Figure 3:**
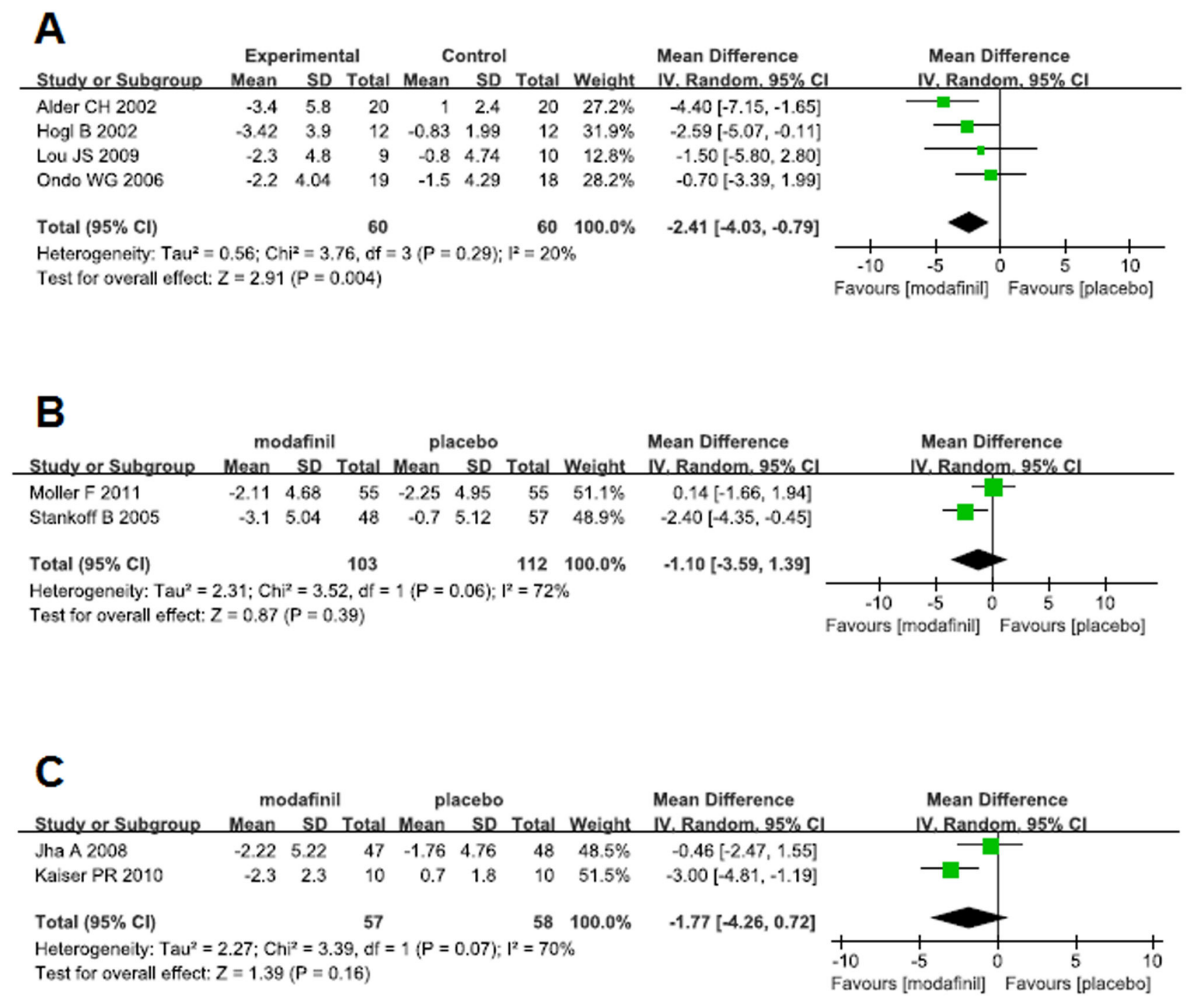
Effects of modafinil on excessive daytime sleepiness measured by Epworth Sleepiness Scale in PD (A), MS (B) and TBI (C).

Subjective measurement of EDS was employed in 4 studies of PD using Epworth Sleepiness Scale (ESS). The overall mean difference was -2.41 (95% CI -4.03 - -0.79) with unimportant heterogeneity (I^2^=20%), demonstrating a clear beneficial effect of modafinil on EDS associated with PD (*p*=0.004) (Figure 3A). The results were not affected by the sensitivity analysis performed by sequentially excluding any study from the main pooled analysis. Moreover, EDS was objectively examined with MSLT in the study by Ondo et al, which didn’t support the beneficial effect of modafinil. No indication of publication bias was observed for studies of PD (Egger’s test, *p*=0.50).

The efficacy of modafinil on EDS in patients with MS was investigated in two large studies. As shown in Figure 3B, beneficial effect of modafinil on EDS was not confirmed in the pooled studies. Likewise, the effect of modafinil on EDS in TBI has been tested in two studies using subjective measures. The study of Jha et al. with a larger number of participants didn’t prove the therapeutic effect of modafinil, while data from Kaiser et al. demonstrated a clear beneficial effect of modafinil on EDS. Meta-analysis of these two studies showed no significant effect of modafinil with a mean difference of -1.77 (95% CI -4.26 - 0.72). The result had a substantial heterogeneity (I^2^=70%) (Figure 3C). Moreover, the study of Kaiser et al. examined EDS with MWT, demonstrating superiority of modafinil versus placebo. The effect of modafinil on EDS in patients with PPS was investigated by Vasconcelos OM et al. Improvements were seen in ESS with both placebo and modafinil with no significant differences between the two treatments [46].

### Efficacy of modafinil on depression associated with neurological disorders

**Figure 4:**
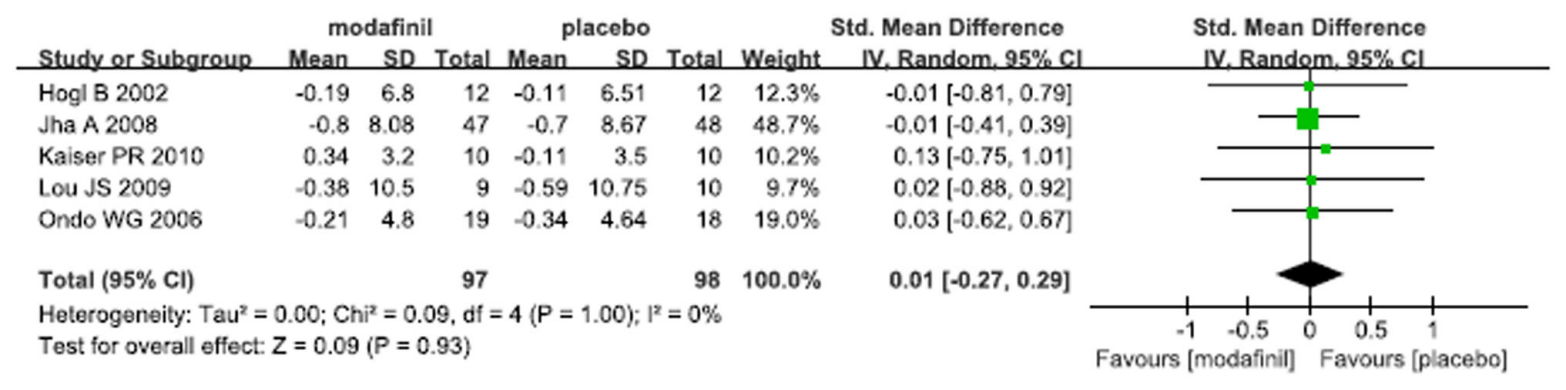
Effect of modafinil on depression associated with neurological disorders.

Five RCTs examining the effect of modafinil on depression associated with neurological disorders came up with consistent outcomes, in which Beck Depression Inventory (BDI) was used in 3 studies, Hamilton Depression Scale (HDS) in one and Center of Epidemiological Study-Depression Scale (CES-D) in another. The pooled standardized mean difference demonstrated no impact of modafinil on depression associated with neurological disorders (SMD 0.01, 95% CI -0.27 - 0.29, p=0.93, I^2^=0%) (Figure 4). There was no significant indication of publication bias (Egger’s test, p=0.542).

### Adverse effects

**Figure 5:**
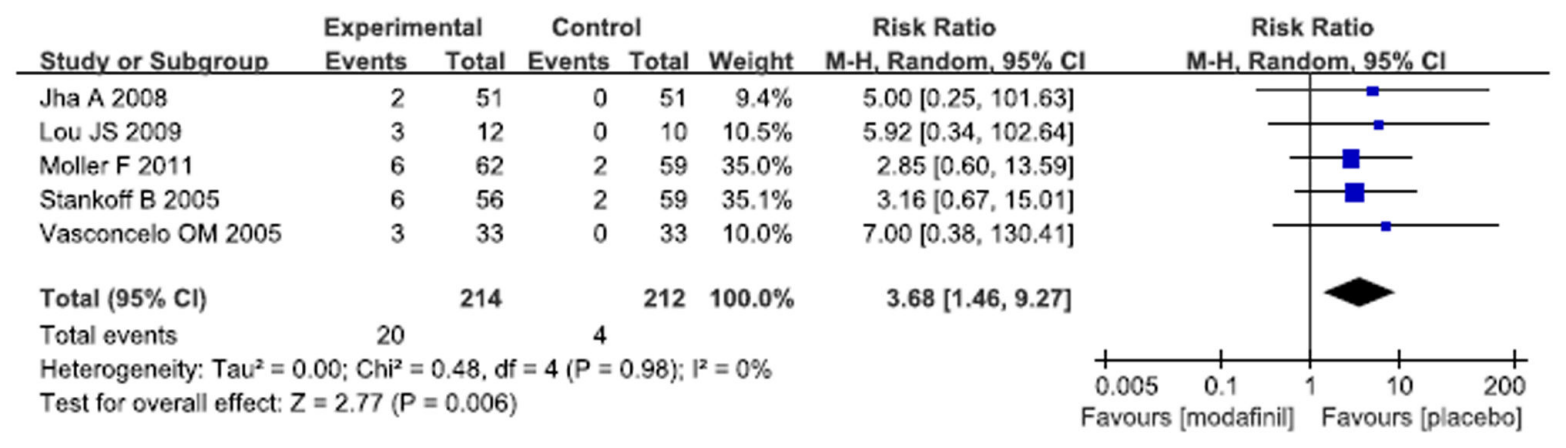
Effect of modafinil on study discontinuation due to adverse events.

Of 10 studies included, the adverse effects were described in 9% of patients in modafinil group and 2% of patients in placebo group. The overall risk ratio for study discontinuation due to side effects suggested that patients treated with modafinil were more likely to withdraw from treatment compared to patients with placebo (RR 3.68, 95% CI 1.46 - 9.27, p=0.006, I^2^=0%) (Figure 5).

Occurrence of adverse events reported in the included studies was summarized in Table 2. Generally, more patients reported insomnia and nausea in modafinil group compared to placebo group. Other rates of adverse events were similar between the two groups.

**Table 2:** The pooled adverse effects of modafinil in included studies.

**Adverse effect**	**No. of studies**	**No. of patients modafinil/placebo**	**Risk Ratio and 95%CI**	***P* value**	**I^2^ (%)**
Insomnia	5	172 / 175	4.20 [1.52, 10.60]	0.002	0
Headache	4	160 / 163	1.19 [0.70, 2.03]	0.53	0
Dizziness	4	138 / 140	2.40 [0.71, 8.15]	0.16	0
Anxiety	3	95 / 97	1.23 [0.23, 6.72]	0.81	0
Nausea	4	136 / 138	3.79 [1.29, 11.16]	0.02	0
Diarrhea	3	108 / 110	1.21 [0.22, 6.59]	0.83	0

## Discussion

EDS and fatigue are highly prevalent symptoms, especially in primary care and specialty medicine. They have overlapping features, which may contribute to imprecise diagnosis and inappropriate treatment. In general, EDS is depicted as drowsiness, sleep propensity and decreased alertness while fatigue is often described as weariness, weakness and depleted energy [49]. Our primary research question was aimed at assessing the effects of modafinil on fatigue and EDS associated with neurological disorders and its safety. This review identified 10 RCTs concerning PD, MS, TBI, and PPS in which a total of 120 patients with PD, 234 with MS, 115 with TBI and 66 with PPS were enrolled. Our results suggested the therapeutic effect of modafinil on fatigue in TBI, with a clear beneficial effect of modafinil on EDS in patients with PD, while the beneficial effect of modafinil on fatigue could not be confirmed in the pooled studies of PD or MS, and the therapeutic effect of modafinil on EDS was not seen in patients with MS and TBI as well. For PPS, improvements were seen in FSS and ESS with both placebo and modafinil without significant differences between the two groups. Modafinil seemed to have no therapeutic effect on depression. In general, adverse events were similar between modafinil and placebo groups except that more patients reported insomnia and nausea in modafinil group compared to the placebo group.

Modafinil is an oral wake-promoting agent, which has been approved by the FDA to improve wakefulness in patients with narcolepsy, OSA and SWSD. Previous meta-analysis suggested a therapeutic effect of modafinil on EDS, but not cataplexy in narcoleptic patients [50]. In that study, EDS was assessed thoroughly by ESS, MSLT, MWT, as well as the number and duration of somnolence, sleep attacks and naps per day. Additionally, their results indicated a beneficial impact of modafinil on quality of life according to the SF-36 questionnaire. In the current study, our data did not demonstrate a consistent effect of modafinil on EDS in the neurological disorders. Superior effect estimate was observed only in PD, but not in MS and TBI. Of note, patients with PD in 3 of 4 studies sustained relatively higher ESS scores, compared to patients with MS and TBI, indicating more severe EDS on these patients. The mean ESS scores were 14.46 for PD, 10.97 for MS and 8.64 for TBI. The ESS is an 8-item questionnaire intended to measure daytime sleepiness with a score between 0 and 24. A number ranged 0 to 9 is considered as normal, while a number ranged 10 to 24 indicates excessive daytime sleepiness [51]. The mean ESS scores for patients with TBI and participants with MS were within or a bit above a normal range respectively. Hence, a floor effect might have been observed in the studies of MS and TBI. Additionally, the studies of Högl et al. and Kaiser et al. employed both subjective (ESS) and objective (MSLT/MWT) measures. Kaiser et al. came to a consistent conclusion based on MWT and ESS tests, while data from MSLT didn’t match with ESS result in the study of Högl et al. It has been reported that there is no statistical and clinical association between ESS and MLST [52]. The subjective ESS and objective MSLT/MWT might probably evaluate different, complementary aspects of sleepiness. EDS can be a symptom of a number of factors and disorders, i.e. poor night sleep, shift work, OSA, restless legs syndrome and depression. OSA is a major public health problem and is very prevalent in patients with neurological disorders [19,20,52]. A standard diagnosis of OSA requires polysomnographical investigations. However, in the studies included, few patients underwent polysomnographical investigations to identify persistent sleep disorders such as OSA, which can be a major confounder in the interpretation of the effect of modafinil on EDS associated with neurological disorders. Hence, polysomnographical investigations are needed to screen out patients with OSA and it will be probably useful to exclude patients with sleep disorders from randomized trials on modafinil in the future to avoid a confounder.

Besides EDS, fatigue is another frequent complaint among patients with neurological disorders. Currently fatigue is identified by the response to a single item on a general health questionnaire or from one or two symptom criteria from symptom checklists [53]. FSS is a one-dimensional fatigue measure and MFIS is a short, multidimensional subjective fatigue measure. They are both employed in the studies of MS. In order to avoid introduction of possible heterogeneity into the results, we didn’t combine results from RCTs with different instruments together. The meta-analyses of fatigue, examined by either FSS or MFIS, did not show beneficial effect of modafinil. Our data were in accordance with the Cochrane review by Peuckmann-Post V et al. [54]. In our study, we pooled two studies to evaluate the effect of modafinil on fatigue in TBI. Although meta-analysis showed superior effect estimate for modafinil in TBI compared to placebo, the result should be interpreted with caution due to a limited number of participants and extremely unbalanced weight of the two studies. Veautheir et al. and Kaminska et al. described a clear and significant relationship between MS-related fatigue and sleep disorders and found an especially strong association between severe fatigue and severe OSA [19,20]. Furthermore, a significant association between severe fatigue and the respiratory arousal index was seen in MS patients, suggesting that respiratory-related sleep fragmentation rather than intermittent hypoxemia might be primarily responsible for the increased fatigue [20]. The effects of modafinil on sleep in OSA were conducted with overnight polysomnography in 3 RCTs [55-57]. Sleep efficiency and the architecture of the sleep were not affected by modafinil. The number of arousals was not significantly changed by modafinil in two trials, while the mean arousal index was statistically higher with modafinil than with placebo in the third trial. Although modafinil is proven to be effective in ameliorating EDS in some specific conditions [27,28], it doesn’t improve the respiratory-related sleep fragmentation, which is closely associated with increased fatigue [55-57]. This might be one of the potential explanations why modafinil failed to decrease fatigue in neurological disorders. Owing to the fact that sleep disorders may be one of the causes responsible for neurological disorder related fatigue, it will be probably useful to exclude patients with sleep disorders from modafinil-RCT in the future to avoid a confounder.

Depression is highly associated with fatigue and EDS [58-60]. Previous evidence suggested modafinil in association with antidepressant medication can improve overall clinical condition including depressive symptoms, fatigue and EDS (61,62). Our results indicated that administration of modafinil alone could not ameliorate depressive symptoms, supporting the opinion that modafinil can be regarded as an agent in augmentation therapy of depression. 

There are some limitations in our study. The available data from RCTs are scare although there is a quantity of case reports and uncontrolled trials. It has to be kept in mind that many of the included studies involved only a small number of participants and did not follow a consistent research methodology. Of 10 RCTs, 4 studies were crossover design. It might be unjustified to pool the data of crossover and parallel studies together, which might introduce heterogeneity into the results. Further, assessment of fatigue and EDS were performed with subjective instrument, which greatly depended on participant’s cognitive ability. Patients with neurological diseases often maintain cognitive deficits and are less aware of their problems [63]. Compared to subjective measures, polysomnographical investigations are relatively independent of participant’s cognitive ability. The present review only included two studies where objective measurement of EDS, i.e. MSLT and MWT were employed. As for fatigue, no objective measurement has been developed yet. On the one hand, fatigue is a subjective symptom and can only be assessed subjectively by definition. On the other hand, it is assumed that perceived fatigue correlates with some objective read-outs as e.g. MSLT, MWT or measures of cognitive performance as attention [64]. In fact, combination of endpoints would be the best solution to evaluate disorder related fatigue. This may indicate that the major problem might not be that modafinil does not work, but the lack of capacity to develop sound trial concepts and to homogenize patients with presumably different fatigue pathogenesis. Finally, modafinil in the identified studies was administrated in short-term settings. Despite the fact that no severe adverse events were presented in the current research, the safety of modafinil in the long-term administration, especially the potential of abuse and addiction, need to be investigated in the future trials.

## Conclusion

Existing trials of modafinil for fatigue and EDS associated with Parkinson’s disease, multiple sclerosis, traumatic brain injury and post-polio syndrome provided inconsistent results. The majority of the studies had small sample sizes. Modafinil is not yet sufficient to be recommended for these medical conditions until solid data are available. It would be ideal to perform large RCTs in MS and PD investigating the effect of modafinil on either fatigue or sleepiness and sleep disorders should be excluded as a major confounder by polysomnography in these studies.

## Supporting Information

Checklist S1 **PRISMA checklist.**
(DOC)

File S1 **Combination of Key Words Used in the Literature Search.**
(DOC)

Flowchart S1 **PRISMA Flowchart.**
(DOC)
