# Supplementary material for: Efficacy of Modafinil on Fatigue and Excessive Daytime Sleepiness Associated with Neurological Disorders: A Systematic Review and Meta-Analysis
Source: PLoS One. 2013 Dec 3;8(12):e81802. doi: 10.1371/journal.pone.0081802 (PMC3849275; doi:10.1371/journal.pone.0081802)
Supplement: File S1 — Combination of Key Words Used in the Literature Search. (DOC) [file pone.0081802.s002.doc]

**Supplement S1.** Search Terms

|  | And | And |
| --- | --- | --- |
| brain injury | Fatigue | Modafinil |
| head injury | Asthenia | armodafinil |
| brain trauma | Hypersomnia |  |
| head trauma | Excessive daytime sleepiness |  |
| stroke |  |  |
| brain ischemia |  |  |
| intracranial hemorrhage |  |  |
| cerebral ischemia |  |  |
| cerebrovascular disease |  |  |
| Parkinson’s disease |  |  |
| parkinsonism |  |  |
| PD |  |  |
| multiple sclerosis |  |  |
| MS |  |  |
| disseminated sclerosis |  |  |
| Alzheimer’s disease |  |  |
| AD |  |  |
| post-polio syndrome |  |  |
| brain tumor |  |  |
| brain neoplasms |  |  |
| brain cancer |  |  |
| cerebral neoplasms |  |  |
| intracranial neoplasms |  |  |
| glioma |  |  |
| meningioma |  |  |
| pituitary tumor |  |  |
