# Supplementary material for: Efficacy of Modafinil on Fatigue and Excessive Daytime Sleepiness Associated with Neurological Disorders: A Systematic Review and Meta-Analysis
Source: PLoS One. 2013 Dec 3;8(12):e81802. doi: 10.1371/journal.pone.0081802 (PMC3849275; doi:10.1371/journal.pone.0081802)
Supplement: Flowchart S1 — PRISMA Flowchart. (DOC) [file pone.0081802.s003.doc]

**
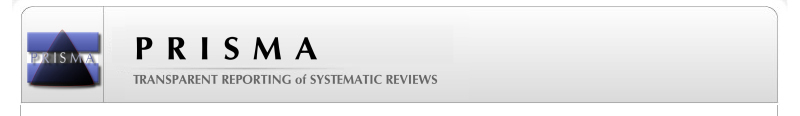
PRISMA 2009 Flow Diagram**

**Screening**

**Included**

**Eligibility**

**Identification**

**Records identified**

Embase (n=410)

Medline (n =59)

Cochrane (n=14)

PSYCHInfo (n=6)

Records after duplicates removed (n =427)

Records screened (n =89)

**Records excluded (n =338)**

Not original data (n=309)

Case report (n=29)

Full-text articles assessed for eligibility (n =92)

**Full-text articles excluded (n =82)**

Not original data (n=42)

Patients without concerned diseases (n=2)

Not about modafinil (n=13)

Without control group (n=20)

Without interest outcome (n=3)

Without valid data (n=2)

**Studies included in meta-analysis (n =10)**

Articles selected from reference lists of retrieved articles (n =3)
